# Improving early recognition and intervention in people at increased risk for the development of bipolar disorder: study protocol of a prospective-longitudinal, naturalistic cohort study (Early-BipoLife)

**DOI:** 10.1186/s40345-020-00183-4

**Published:** 2020-07-01

**Authors:** Andrea Pfennig, Karolina Leopold, Julia Martini, Anne Boehme, Martin Lambert, Thomas Stamm, Felix Bermpohl, Andreas Reif, Sarah Kittel-Schneider, Georg Juckel, Andreas J. Fallgatter, Tilo Kircher, Andreas Jansen, Steffi Pfeiffer, Christina Berndt, Maren Rottmann-Wolf, Cathrin Sauer, Philipp Ritter, Christoph U. Correll, Andreas Bechdolf, Irina Falkenberg, Michael Bauer

**Affiliations:** 1 Department of Psychiatry and Psychotherapy, Carl Gustav Carus University Hospital, Technische Universität Dresden, Fetscherstraße 74, 01307 Dresden, Germany; 2 Department of Psychiatry, Psychotherapy and Psychosomatics, Vivantes Klinikum Am Urban, Berlin, Germany; 3 Department of Psychiatry and Psychotherapy, University Medical Center Hamburg-Eppendorf, Hamburg, Germany; 4 Department of Psychiatry and Psychotherapy, Charité Universitätsmedizin Berlin, Berlin, Germany; 5 Department of Psychiatry, Psychotherapy and Psychosomatic, Medical School Brandenburg, Neuruppin, Germany; 6 Department of Psychiatry, Psychosomatics and Psychotherapy, University Hospital Frankfurt, Frankfurt, Germany; 7 Department of Psychiatry and Psychotherapy, LWL-University Hospital Bochum, Ruhr-University Bochum, Bochum, Germany; 8 Department of General Psychiatry and Psychotherapy, University Hospital Tübingen, Tübingen, Germany; 9 Department of Psychiatry and Psychotherapy, University Hospital Marburg, Marburg, Germany; 10 The Donald and Barbara Zucker School of Medicine at Hofstra/Northwell, The Zucker Hillside Hospital, Glen Oaks, NY USA; 11 Department of Child- and Adolescent Psychiatry, Charité Universitätsmedizin Berlin, Berlin, Germany

**Keywords:** Bipolar disorder, Early recognition, Early intervention, Risk factor, Prevention, Antecedent, Precursor, Protective, Resilience

## Abstract

**Background:**

Bipolar disorders (BD) belong to the most severe mental disorders, characterized by an early onset and recurrent, severe episodes or a chronic course with poor psychosocial functioning in a proportion of patients. Many patients with BD experience substantial symptomatology months or even years before full BD manifestation. Adequate diagnosis and treatment is often delayed, which is associated with a worse outcome. This study aims to prospectively evaluate and improve early recognition and intervention strategies for persons at-risk for BD.

**Methods:**

Early-BipoLife is a prospective-longitudinal cohort study of 1419 participants (aged 15–35 years) with at least five waves of assessment over a period of at least 2 years (baseline, 6, 12, 18 and 24 months). A research consortium of ten university and teaching hospitals across Germany conducts this study. The following risk groups (RGs) were recruited: RG I: help-seeking youth and young adults consulting early recognition centres/facilities presenting ≥ 1 of the proposed risk factors for BD, RG II: in-/outpatients with unipolar depressive syndrome, and RG III: in-/outpatients with attention-deficit/hyperactivity disorder (ADHD). The reference cohort was selected from the German representative IMAGEN cohort. Over the study period, the natural course of risk and resilience factors, early symptoms of BD and changes of symptom severity (including conversion to manifest BD) are observed. Psychometric properties of recently developed, structured instruments on potential risk factors for conversion to BD and subsyndromal symptomatology (Bipolar Prodrome Symptom Scale, Bipolar at-risk criteria, EPI*bipolar*) and biomarkers that potentially improve prediction are investigated. Moreover, actual treatment recommendations are monitored in the participating specialized services and compared to recently postulated clinical categorization and treatment guidance in the field of early BD.

**Discussion:**

Findings from this study will contribute to an improved knowledge about the natural course of BD, from the onset of first noticeable symptoms (precursors) to fully developed BD, and about mechanisms of conversion from subthreshold to manifest BD. Moreover, these generated data will provide information for the development of evidence-based guidelines for early-targeted detection and preventive intervention for people at risk for BD.

## Background

Bipolar disorder (BD) is a severe and disabling mental disorder, characterized by an early onset and recurrent, severe episodes or a chronic course with poor psychosocial functioning in a proportion of patients (Ferrari et al. 2016; Fagiolini et al. 2013). Although efficacious psychopharmacological and psychosocial treatments for manifest BD exist (da Silva Lima et al. 2017; Pfennig et al. 2012a), BD is often diagnosed and treated with a significant delay (up to 10 years on average) (Baldessarini et al. 2003; Pfennig et al. 2011; Dagani et al. 2017). Treatment delay is associated with a worse functional outcome, an elevated risk of suicide (Chen and Dilsaver 1996; Miller et al. 2014; Post et al. 2010) and an inferior response to mood stabilizing drug treatment (Kessing et al. 2014). Improved diagnostic instruments for early recognition and guidelines for early-targeted intervention have the potential to enhance overall disease management or even to prevent conversion to manifest BD (Lish et al. 1994).

Recent evidence from early recognition centers has shown that help-seeking persons at-risk for BD are often already affected by a substantial impairing subsyndromal symptomatology (Leopold et al. 2013a, 2014; Pfennig et al. 2012b). Given the low specificity of individual precursors and early symptoms of BD prior to the first manifest manic episode (Berk et al. 2007), comprehensive criteria defining high-risk profiles need to be assembled. Due to its high heritability (Craddock and Sklar 2009), a positive family history for BD is one of the major risk factors for BD (Duffy et al. 2010, 2014; Smoller and Finn 2003; Mendlewicz and Rainer 1977). Moreover, evidence from prospective clinical and observational studies indicates that depressive and subthreshold (hypo-)manic symptoms may be antecedents or rather first noticeable symptoms of BD (Beesdo et al. 2009; Duffy et al. 2014, 2017; Bechdolf et al. 2012; Mesman et al. 2013; Ryles et al. 2017).

There is a vivid discussion about the association between attention-deficit/hyperactivity disorder (ADHD) and BD. At least a subgroup of patients with ADHD may be at risk to develop BD (Leopold et al. 2012; Faedda et al. 2014; Wang et al. 2016). Additionally, the following factors are discussed as further potential risk factors for BD: (a) a history of critical/stressful life events (Garno et al. 2005; Kessing et al. 2004), (b) childhood anxiety disorders or being anxious/worried/fearful, hyper-alert or sensitive (Lenox et al. 2002; Egeland et al. 2003; Tillman et al. 2003), (c) mood swings and impaired emotional regulation (frequent “ups and downs”) (Thompson et al. 2003), (d) changes in sleep and circadian rhythm (Lenox et al. 2002), (e) somatic complaints or medical/physical problems (Egeland et al. 2003), (f) substance (ab-)use or dependence (Rush 2003), and (g) particular personality, temperament and character traits (e.g., extraversion, novelty seeking, creativity, high reward responsiveness and ambitious goal-striving, cyclothymic/hyperthymic temperament) (Duffy et al. 2010; Mesman et al. 2013; Leopold et al. 2012; Pfennig et al. 2017; Egeland et al. 2000; Correll et al. 2014a; Alloy et al. 2012; Kwapil et al. 2000; Blechert and Meyer 2005).

Based on this knowledge, structured instruments have been developed by different research groups to identify persons at risk for BD (Leopold et al. 2012; Bechdolf et al. 2014; Correll et al. 2014b) and to assess initial subsyndromal symptomatology (Bipolar Prodrome Symptom Scale, Correll et al. 2014b), EPI*bipolar*, Leopold et al. 2012), Bipolar at-risk criteria (Bechdolf et al. 2012)/extended BAR criteria (Fusar-Poli et al. 2018). First prospective studies revealed promising results on the psychometric properties of single instruments and the predictive validity regarding the conversion to BD (Bechdolf et al. 2014; Correll et al. 2014b; Fusar-Poli et al. 2018). Bechdolf and colleagues showed a conversion rate to BD of 14.3% within 12 months in people presenting with BAR criteria (Bechdolf et al. 2014), and Birmaher and colleagues found a conversion rate of 25% to BD in children and adolescents with clinically relevant bipolar symptoms that did not fulfil the DSM-IV criteria for bipolar-I or bipolar-II disorder (Birmaher et al. 2006). Conversion rates of patients with unipolar depression range in different studies from 4 to 49% (Geller et al. 2001; Gilman et al. 2012). However, previous samples were small, and the comparison of study results is hampered by heterogeneous risk definition/description and differences in study methodology (e.g., diverse follow-up duration). Standardization of the diagnostic process across specialized early recognition facilities is not formed yet. Moreover, at present protective/resilience factors (Stange et al. 2013) and potential biomarkers (Duffy et al. 2014; Ritter et al. 2016; Kapczinski et al. 2009) are not part of the existing assessment tools (Leopold et al. 2012; Bechdolf et al. 2014; Correll et al. 2014b).

Besides the challenges of early and appropriate diagnostics, prevention as well as early-targeted intervention of BD is crucial, because treatment delay is associated with a worse social adjustment, an increased risk for suicide, increased comorbidity rates, more hospitalizations, and an impaired age-appropriate development (Goldberg and Ernst 2002; Matza et al. 2005). Pharmacological treatment approaches with mood stabilizing agents (lithium and divalproex) in at-risk patients have only been investigated in underpowered studies not showing effectiveness (Findling et al. 2007; Geller et al. 1998). Regarding monotherapy with antidepressants, study data in bipolar at-risk patients are lacking, and there might be a risk for inducing mania. However, in case of depressive, anxiety and/or obsessive symptomatology, antidepressant treatment under close monitoring is in line with guideline recommendations for both, unipolar (DGPPN 2015) and bipolar (DGBS and DGPPN 2019) disorders. A recent review of psychotherapeutic interventions in young at-risk patients (Pfennig et al. 2017) showed promising results. Three studies on the efficacy of early family-focused approaches suggest favorable outcomes such as improved symptoms, a longer duration in remission and a better psychosocial functioning (Miklowitz et al. 2011, 2013; Nadkarni and Fristad 2010). A updated systematic review (Blum 2018) provided evidence for the efficacy of Interpersonal and social rhythm therapy (IPSRT, Goldstein et al. 2018) and Mindfulness-based cognitive therapy (MBCT, Cotton et al. 2016) with improvement in sleep patterns, emotion regulation and a decrease of anxiety, compared to baseline.

Based on the knowledge of risk factors and early clinical features of BD and of the promising research on early targeted interventions, pilot clinical category models and treatment guidance (Berk et al. 2007; Kapczinski et al. 2009; Leopold et al. 2013b; Schneck et al. 2017) have been formulated. Primary treatment recommendations for early (at-risk) stages of BD are psychoeducation, preventive strategies to halt conversion to BD, and strategies on preserving the young person’s ability to meet age-appropriate developmental tasks (Berk et al. 2007; Kapczinski et al. 2009; Leopold et al. 2013b). At later stages, the focus changes to symptomatic treatment, and the establishment of adherence and relapse prevention of BD (Berk et al. 2007; Kapczinski et al. 2009; Leopold et al. 2013b). The verification of the suggested models and guidance could enable evidence-based treatment approaches in early stages of BD. A validated model would provide greater utility for testing the efficacy, cost-effectiveness, risk–benefit ratios and feasibility of available interventions (McGorry et al. 2006).

In summary, early recognition of at-risk states for BD is an important clinical field, but the diagnostic process across specialized early recognition facilities has not been standardized yet. First approaches of clinical categorization and treatment guidance have been formulated (Berk et al. 2007; Kapczinski et al. 2009; Leopold et al. 2013b), but no common diagnostic and/or treatment guidelines are available supporting the individual decision-making processes for those in the early stages of BD. This situation impedes the accumulation of superior clinical-epidemiological knowledge regarding the core predictors for conversion to manifest BD and prevention of incident BD.

To address these areas of unmet needs, a prospective, naturalistic cohort study in the age group of 15–35 years was designed to clarify the following research questions, with (a) being the primary question:

a What is the predictive power of the individual risk factors/constellations in defined risk groups for BD using the existing instruments and recommended at-risk criteria? What is the prevalence of these risk factors and constellations in a representative cohort? How do risk factors interact?
b What are protective/resilience factors for BD in the proposed age group?
c What is the association of biomarkers with the clinical outcome?
d How can the information from (a), (b), and (c) be integrated for further development of existing diagnostic tools for early detection (interviews, risk criteria) and standardization/harmonization of the diagnostic process across centres/facilities?
e What factors are relevant for treatment decision-making in this naturalistic setting?
f What information from (a–e) can be used to refine existing category model and treatment guidances?

## Methods

The *Improving early recognition and intervention in people at*-*risk for development of bipolar disorder* (*Early*-*BipoLife*) study is a multicentre study conducted by a research consortium of ten university and teaching hospitals across Germany with early recognition centres/facilities and specialised in- and outpatient care. *Early*-*BipoLife* is funded by the Federal Ministry of Education and Research (BMBF, Grant number: 01EE1404A) and is part of the BipoLife consortium described elsewhere (Ritter et al. 2016; Mühlbauer et al. 2018). The study is conducted according to good clinical practice (GCP) standards and has been approved by the responsible Ethics Committee of the Medical Faculty of the Technische Universität Dresden (No: EK290082014) and all local ethics committees. All participants provided written informed consent after comprehensive information about study aims and procedures.

### Study design and procedures

Early-BipoLife is a naturalistic, prospective-longitudinal observational cohort study of participants aged 15–35 years with at least five waves of assessment over a period of at least 2 years.

From July 2015 until September 2018, help seeking youth and young adults consulting early recognition centres/facilities presenting with at least one of the proposed risk factors for BD as well as in- and outpatients with depressive syndrome or ADHD, respectively, were recruited at the network sites (in Berlin (two sites), Bochum, Dresden, Frankfurt/Main, Hamburg, Marburg, Brandenburg/Neuruppin, Tübingen).

The reference cohort was selected from the German representative IMAGEN cohort (Berlin, Dresden). This cohort is a representative population-based sample of N = 2000 young people from Germany, the United Kingdom, Ireland and France who were recruited at the age 14 years with follow-up assessments at 16 and 18 to 20 years (https://imagen-europe.com). For the Early-BipoLife study assessment, subjects from the IMAGEN cohort were recruited from July 2016 to December 2018. With their informed consent, data from the previous assessments at age 14 and 16 will be used in the present study analyses.

Participants were examined at baseline (BL), and after 6 months (FU1), 12 months (FU2), 18 months (FU3) and 24 months (FU4) with further long-term follow-up (currently up to month 36). At BL, FU2 and FU4, participants are assessed with a comprehensive standardized face-to-face diagnostic procedure by trained academic study personnel (physicians and psychologists). The mean time for completion of the standardized interview and additional questionnaires is approximately 3 to 7 h (mostly conducted over three appointments). FU1 and FU3 are conducted as telephone-interviews with a duration of approximately 30 min. If indicators for change in risk score severity or conversion to BD are registered in the telephone interview, a face-to-face contact with the comprehensive assessment battery is conducted. Interviewers/raters at baseline and follow-up were trained centrally in a 2-day training before starting to recruit at the individual study center, were supervised locally by the principal investigator of the respective study center and were re-trained regularily. Reliability was assessed for the early detection instruments and the SCID. FU-raters were not blinded for risk status. The risk detection instruments use historical information and change to former FU, so to ensure to rate the current risk profile accurately, using historical information was allowed.

**Fig. 1:**
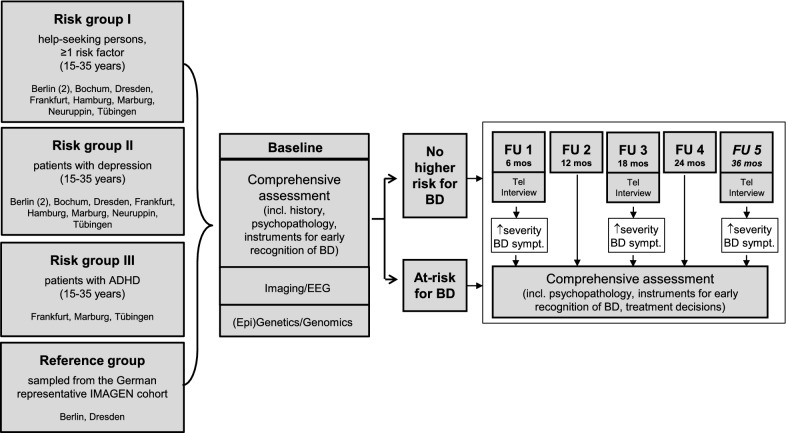
Design of the BipoLife-study

The design and procedures of the Early-BipoLife study are displayed at Fig. 1.

Participants further receive the option to participate in the neuroimaging, electroencephalography (EEG), and genomics platform projects within the Early-BipoLife study. Details of the used methods and paradigms are described below and elsewhere (Ritter et al. 2016).

Since individuals not (yet) meeting BD diagnostic criteria may benefit from a structured and supervised diagnostic and decision-making process with tailored symptomatic treatment, all persons in RG I (see below) receive state-of-the-art counselling and treatment according to their individual needs. Although clinical category models and treatment guidance were recently postulated (Berk et al. 2007; Leopold et al. 2013b), it is not clear to what extent those theoretical models are implemented in daily care and how beneficial they are to the individual patient. Due to the naturalistic design of the Early-BipoLife study, the content and the extent of the particular counselling and treatment recommendations are at the discretion of the individual study centre staff and reflect the particular centre’s usual care that is based on the clinical experience of the site’s clinical experts (Pfennig et al. 2012b). Particular recommendations (e.g., general or specific preventive strategies, psychotherapy, and/or pharmacotherapy) are monitored and analysed during the Early-BipoLife study. A comparison with the recently postulated models and guidance will be conducted (Berk et al. 2007; Kapczinski et al. 2009; Leopold et al. 2013b; Schneck et al. 2017).

### Sampling and initial risk groups

Overall, N = 2279 persons were screened for inclusion and exclusion criteria. For eligibility of participants for the Early-BipoLife study (inclusion and exclusion criteria) see Table 1.

**Table 1:** Key inclusion and exclusion criteria for study participants of the risk groups I–III

	Inclusion criteria	Exclusion criteria
Risk group I (RG I)	• Age: 15 to 35 years • Consultation of an early recognition centre/facility • Presence of at least one of the proposed risk factors for BD: – Family history of BD – (Sub)threshold affective symptomatology/depressive syndrome – Hypomanic/mood swings – Disturbances of circadian rhythm/sleep other clinical hints	• Diagnosis of: BD, schizoaffective disorder, schizophrenia • Diagnosis of anxiety, obsessive–compulsive or substance dependence disorder that fully explains the whole symptomatology • Limited ability to comprehend the study • Implied expressed negative declaration of intent to participate in the study by a minor and • Acute suicidality
Risk group II (RG II)	• Age: 15 to 35 years • In- or outpatients with a depressive syndrome in the context of: – Major depressive disorder – Dysthymic disorder – Cyclothymic disorder – Minor depressive disorder – Recurrent brief depressive disorder – Adjustment disorder with depressed mood – Depressive disorder Not Otherwise Specified (NOS)	
Risk group III (RG III)	• Age: 15 to 35 years • In- or outpatients with a clinically confirmed ADHD diagnosis	

In total, N = 1419 study participants were included in the study. Due to their baseline diagnostic status, risk participants (N = 1229) were assigned to one of the above mentioned risk groups (RG I–III).

### Measures

Core instruments to observe and predict the natural course of disease in terms of change of severity of the risk status are the EPI*bipolar* (Leopold et al. 2012), the BPSS-P (Correll et al. 2014b) and the BAR criteria (Bechdolf et al. 2012)/extended BAR criteria (Fusar-Poli et al. 2018) (see “Core instruments” below). The assessments further include interviewer ratings and self-report scales to characterize the development of the risk constellation during the study period and to examine disease progression from subthreshold and threshold symptomatology of BD [EPI*bipolar* (Leopold et al. 2012), BPSS-P (Correll et al. 2014b), BAR (Bechdolf et al. 2012)/extended BAR criteria (Fusar-Poli et al. 2018), SCID-I (Wittchen et al. 1997), IDS-C (Drieling et al. 2007), QIDS–SR16 (Roniger et al. 2015), YMRS (Mühlbacher et al. 2011), ASRM (Bernhard and Meyer 2011)].

The primary outcome of the study is change in illness severity in terms of (a) change of the risk status in the core instruments, (b) initial prescription of a drug with the aim of mood stabilization, (c) conversion to manifest BD, or (d) change in clinical relevant burden of disease or impairment of psychosocial functioning [measured with EPI*bipolar* (Leopold et al. 2012), GAF (Hall 1995), FAST (Riegler et al. 2017; Rosa et al. 2007) and WHOQOL-BREF (Angermeyer et al. 2000)].

Throughout the study, comorbid disorders (SCID-I, Wittchen et al. 1997; SCID-II, Wittchen et al. 1997), psychiatric treatment, physical illness, substance use (Case Report Form, CRF) and symptoms of psychotic prodrome (PQ-16, Ising et al. 2012; SIPS/SOPS, Miller et al. 2003; SPI-A, Schultze-Lutter et al. 2012) are monitored.

There is agreement that resilience is not equal to absence of risk. Resilience has been described as the process of adapting well in the face of adversity, trauma, threats, or significant stress (American Psychological Association 2014). Potential protective/resilience factors assessed include sociodemographic variables (e.g., living with a partner, being employed), resources and self-management skills (measured with FERUS, Jack 2004) as well as help-seeking behaviour (CRF). These are then analysed in relation to perceived stress, stressful life-events and psychosocial functioning.

### Core instruments

Three recently developed structured instruments for potential risk factors/constellations for conversion to BD and initial subsyndromal symptomatology are applied in parallel throughout the study.

The *Bipolar Prodrome Symptom Scale*—*Prospective* (BPSS-P, © Correll 2013, Correll et al. 2014b) is a semi-structured interview developed based on the DSM-IV criteria for BD and Major Depressive Disorder (MDD) as well as established rating scales for symptoms of the manic, depressive and psychotic spectrum. Moreover, recent literature on risk factors for BD was considered. The BPSS-P assesses the onset and severity of prodromal symptoms in three sections (Mania Symptom Index, Depression Symptom Index, and General Symptom Index). The BPSS-P has good internal consistency, convergent validity and inter-rater reliability (Correll et al. 2014b).

*Bipolar at*-*risk criteria* (BAR criteria, © Bechdolf 2012, Bechdolf et al. 2012): Bechdolf and colleagues developed a set of ultra-high-risk criteria for BD that comprise sub-threshold clinical and behavioral information as well as genetic risk. BAR criteria are met when persons are aged 15–24 years and fulfil the criteria of at least one of the following at-risk groups: sub-threshold mania (Group I), depression plus cyclothymic features (Group II), depression plus genetic risk (Group III). One prospective study revealed promising results regarding the predictive validity of the BAR criteria for conversion to manifest BD (Bechdolf et al. 2012, 2014). The extension of the BAR criteria proposed by the group of Irina Falkenberg was assessed adding the at-risk groups mixed symptoms (Group IV) and mood swings (Group V).

The *Early Phase Inventory for Bipolar Disorders* (EPI*bipolar*, © Pfennig and Leopold 2012, Leopold et al. 2012) captures risk factor categories that have been identified through a systematic review of the literature and clinical experience. It additionally integrates information from patient’s history, SCID and BPSS. EPI*bipolar* includes the family history of BD, subsyndromal symptomatology (e.g., subthreshold depressive and (hypo-)manic symptoms) and further proposed risk factors for BD (e.g., substance misuse, a diagnosis of ADHD or behavioral problems/conduct disorder, pronounced creativity, critical life events, changes in sleep/circadian rhythm, mood swings or increased affective lability, fearfulness/anxiety, dissociative symptoms, and impairment in psychosocial functioning, Leopold et al. 2012). Based on this information, risk states for conversion to BD are proposed (risk, high-risk, ultra-high risk).

### Predictors and potential risk and resilience factors for change in severity of BD

Diagnostic and dimensional instruments are listed in Table 2. The following overview operationalizes predictors or potential risk factors for change in the observational outcomes.

**Table 2:** Overview of main instruments and assessment waves

Constructs and instruments	Self-report (SR)/interviewer rating (IR)	Baseline	FU1 6 months	FU2 12 months	FU3 18 months	FU4 24 months	FU5 36 months
Core instruments for early recognition of bipolar disorders							
Early Phase Inventory for bipolar disorders [EPI*bipolar* (Leopold et al. 2012)]	IR	x	(x)^a^	x	(x)^a^	x	(x)^a^
Bipolar Prodrome Symptom Scale—Full Prospective [BPSS-FP (Correll et al. 2014b)]	IR	x	(x)^a^	x	(x)^a^	x	(x)^a^
Bipolar at-risk Criteria [BAR-Criteria (Bechdolf et al. 2012)]	IR	x	(x)^a^	x	(x)^a^	x	(x)^a^
Patient history and mental disorders (DSM, ICD)							
Case Report Form (CRF): study tailored questions on family history of BD, age, sex, marital status, family of origin, housing situation, level of education, employment status and nationality	IR	x		x		x	
Patient history	IR		x	x	x	x	x
Structured Clinical Interview for DSM-IV-TR Disorders [SCID-I (Wittchen et al. 1997)]	IR	x	(x)^a^	x	(x)^a^	x	(x)^a^
SCID-II Screening, in case of positive screening Structured Clinical Interview for DSM-IV-TR Axis II Personality Disorders [SCID-II (Wittchen et al. 1997)]	IR	x					
Telephone interview, study tailored questions on symptoms of BD	IR		x		x		x
Further instruments for the assessment depressive and manic symptoms							
Inventory of Depressive Symptomatology-Clinician [IDS-C (Drieling et al. 2007)]	IR	x					
Quick Inventory of Depressive Symptomatology [QIDS-SR16 (Roniger et al. 2015)]	SR	x					
Young Mania Rating Scale [YMRS (Mühlbacher et al. 2011)]	IR	x					
Altman Self-Rating Mania Scale [ASRM (Bernhard and Meyer 2011; Altman et al. 1997)]	SR	x					
Psychotic features							
PQ-16 Screening, in case of ≥ 6 points: Structured Interview for Prodromal Syndroms, German Version [SOPS (Miller et al. 2003)], Schizophrenia Proneness Interview—Adult version [SPI-A (Schultze-Lutter et al. 2012)]	IR	x					
Temperament							
Barratt Impulsiveness Scale [BIS (Preuss et al. 2008)]	SR	x					
Temperament Evaluation of Memphis, Pisa, Paris and San Diego—Autoquestionnaire short version [TEMPS-A (Victor et al. 2006)]	SR	x					
Behavioral Inhibition System and Behavioral Activation System [BIS/BAS (Strobel et al. 2001)]	SR	x					
Creativity							
Barron Welsh Art Scale [BWAS (Welsh and Barron 1949)]	SR	x					
Creative Achievement Questionnaire [CAQ (Carson et al. 2005)]	SR	x					
Life events and stress							
Childhood Trauma Questionnaire [CTQ (Wingenfeld et al. 2010)] and previous life events (study tailored)	SR	x					
Modified Life Events Questionnaire [MLEQ (McLean et al. 2014)]	SR	x	(x)^a^	x	(x)^a^	x	(x)^a^
Trier Inventory for Chronic Stress [TICS (Schulz et al. 2004)]	SR	x					
Psychosocial functioning and quality of life							
Functioning Assessment Short Test [FAST (Riegler et al. 2017; Rosa et al. 2007)]	IR	x	(x)^a^	x	(x)^a^	x	(x)^a^
Global Assessment of Functioning Scale [GAF (Hall 1995)]	IR	x	(x)^a^	x	(x)^a^	x	(x)^a^
World Health Organization Quality of Life [WHOQOL-BREF (Angermeyer et al. 2000)]	SR	x	(x)^a^	x	(x)^a^	x	(x)^a^
Resources and resilience							
Questionnaire for resources and self-management skills [FERUS (Jack 2004)]	SR	x					

^a^If indicators for change in risk score severity of risk status or conversion to BD are registered in the telephone interview, a face-to-face contact with the comprehensive assessment is conducted

#### Potential risk factors

- Genetic risk: positive family history (1st or 2nd degree relative with a confirmed diagnosis of BD, major depressive disorder, schizoaffective disorder or schizophrenia) (CRF).
- At least subthreshold affective symptomatology (BPSS-P, Correll et al. 2014b), BAR criteria (Bechdolf et al. 2012), SCID-I (Wittchen et al. 1997).
- Mood swings and affective lability (EPI*bipolar*, Leopold et al. 2012).
- Lifetime and present ADHD or conduct disorder (patient’s history; EPI*bipolar*, Leopold et al. 2012).
- Recurrent anxiety (lifetime or present), independent of depressive episodes (EPI*bipolar*, Leopold et al. 2012).
- Specific sleep and circadian rhythm disturbances (EPI*bipolar*, Leopold et al. 2012).
- Substance misuse related to mood swings or affective disturbances (EPI*bipolar*, Leopold et al. 2012).
- Pronounced creativity (BWAS, Welsh and Barron 1949; CAQ, Carson et al. 2005).
- Stressful life events (CTQ, Wingenfeld et al. 2010); section on post-traumatic stress disorder in the SCID-I, Wittchen et al. 1997).
- Affective temperaments (TEMPS-A, Victor et al. 2006).
- Impulsivity (BIS, Preuss et al. 2008).
- Sensitivity of behavioural inhibition/activation system (BIS/BAS, Strobel et al. 2001).
- Chronic stress (TICS, Schulz et al. 2004).
- Psychosocial functioning (before, during and following a symptomatic episode) (EPI*bipolar*, Leopold et al. 2012) and functional impairment (GAF, Hall 1995; FAST, Riegler et al. 2017; Rosa et al. 2007).

#### Further potential influencing factors

- Sociodemographic factors (including age, sex, marital status, level of education and employment status) (CRF).
- Physical health: somatic diseases, BMI (CRF).
- Resources, resilience and self-management skills (FERUS, Jack 2004).
- Stressor load (MLEQ, McLean et al. 2014)
- Help-seeking behaviour (CRF).
- Quality of life (WHOQOL-BREF, Angermeyer et al. 2000).

### Observational outcomes: change in severity

Diagnostic and dimensional instruments listed in Table 2 operationalize observational outcomes of this study as follows:

1. Change in severity of risk status: This criterion is met when there is an increase from a lower to a higher risk state (EPI*bipolar*, Leopold et al. 2012), or if the subject scores on more criteria than in the prior assessments (BPSS-P, Correll et al. 2014b; BAR, Bechdolf et al. 2012/extended BAR criteria, Fusar-Poli et al. 2018).
2. Initial prescription of a drug with the aim of mood stabilization: This criterion is fulfilled when one of the recommended mood-stabilizing agents of the German S3 guidelines on diagnostics and therapy of BD (Pfennig et al. 2012a) is initiated with the purpose of mood stabilization (and not solely for the treatment of depression, prevention of recurrent depressive episodes, or to address insomnia).
3. Conversion to manifest BD: A consensus diagnosis of BD (BD-I or BD-II according to DSM-IV criteria, Saß et al. 2012) is based on the information of the SCID-I (Wittchen et al. 1997) and the confirmation by a consensus board of clinically experienced staff members (psychiatrist and psychotherapist) of the respective specialized service centers/facilities. Conversion to BD occurs when diagnostic criteria for BD-I or BD-II are fulfilled for the first time during the follow-up period.
4. Change in burden of disease and psychosocial functioning: This outcome is defined using EPI*bipolar* (Leopold et al. 2012), GAF (Hall 1995) and FAST (Riegler et al. 2017; Rosa et al. 2007) as well as measures on quality of life (WHOQOL-BREF, Angermeyer et al. 2000). The criterion is met when there is a clinically relevant change in impairment or quality of life compared to the prior assessment.

#### Biomarkers: neuroimaging, electrophysiology and genetics

All participants of the Early-BipoLife study are invited to participate in the neuroimaging, EEG and genomics platform projects.

##### Neuroimaging: functional magnetic resonance imaging (fMRI)

All participants opting for the neuroimaging part complete an identical test battery including:

- T1-sequence for morphometric analyses.
- Resting-state fMRI sequence.
- Three fMRI activation paradigms:
  - Desire-reason dilemma task using conditioned reward stimuli in different experimental situations, thereby allowing the investigation of subcortical structures of the dopaminergic reward system and their specific functional interactions with prefrontal cortical areas (Diekhof and Gruber 2010).
  - Highly robust emotional face matching paradigm for assessing limbic responsiveness to negative facial expressions (Dannlowski et al. 2012).
  - Cartoon Theory-of-Mind (ToM) task, which robustly activates the ToM network relevant for social cognition, since a dysfunction of the ToM network activated by this task is associated with a genetic risk variant for BD and in relatives of patients with BD (Walter et al. 2011).

For quality assurance, a phantom measurement is performed after each subject to investigate the stability of the magnetic resonance signal.

##### Neurophysiology: electroencephalography (EEG)

The EEG battery focusing on neural synchrony in long-range and local oscillatory responses includes cognitive (choice-reaction tasks), perceptive (Kanizsa figures), and emotional (emotional faces) paradigms (Özerdem et al. 2011).

##### Genetics and biomaterial

A network-wide phenotyping and biobanking platform was implemented in synergy with the German Association for Psychiatry and Psychotherapy and Psychosomatics (DGPPN) and their DGPPN Cohort (Anderson-Schmidt et al. 2013) infrastructure. Genomic analyses including targeted genotyping of candidate regions, exome sequencing will be performed. Therefore, saliva samples were acquired following consortium-wide SOPs and protocols and all 2D barcoded material is stored at two mirrored sites in Goettingen and Wuerzburg for genomic, transcriptomic, and proteomic analyses.

Details of the used methods and paradigms are described elsewhere (Ritter et al. 2016).

### Monitoring of treatment

At the end of the baseline assessment as well as the FU2 and FU4, a case conference was held summarizing the findings (regarding established diagnoses and risk status) and the subsequent counselling and treatment procedures within the CRF. As mentioned above, due to the naturalistic design of the Early-BipoLife study, the content and the extent of the particular counselling and treatment recommendations are at the discretion of the individual study centre staff and depict the particular centre’s usual care that is based on clinical experiences of the site’s clinical experts (Pfennig et al. 2012b). Particular recommendations (e.g., general or specific preventive strategies, psychotherapy, and/or pharmacotherapy) are analysed and compared to the recently proposed models and guidance (Berk et al. 2007; Kapczinski et al. 2009; Leopold et al. 2013b; Schneck et al. 2017).

### Statistical analysis

To compare each of the risk groups (RG I–III) with the reference group concerning each of the four observational outcomes that depict change in severity, univariate analyses are conducted (e.g., t-tests, Chi squared tests and univariate ANOVA). Univariate analyses are used to determine individual predictors of change in severity, too.

A multivariate logistic regression model is constructed for each measure of change in severity to determine risk factor constellations that are predictive of change in severity with each individual factor also being significant within the model. The analyses use a backward selection approach to ascertain variables that have unique predictive associations with change in severity at an initially liberal threshold of p < 0.10. After this, another logistic regression analysis is conducted in which variables found to contribute uniquely to change in severity in the initial series are considered together. Variables that remain significant at p < 0.05 in this analysis are then tested for multiplicative (interaction) effects in relation to change in severity. Finally, the identified risk factor(s) are tested regarding their predictive power, calculating sensitivity, specificity, positive predictive value, negative predictive value, and accuracy (e.g., applying ROC analyses).

In addition to the logistic regression model approach, Cox regression analyses are conducted to compare the four study groups concerning time to onset of change in severity, recognizing that individuals will have variable follow-up-times, which will be censored according to a survival analysis approach.

In addition to analysing each risk factor and risk factor constellations, the psychometric properties of the EPI*bipolar*, BPSS-P and BAR criteria are assessed (e.g., Cronbach’s alpha, convergent validity). For all three scales the predictive power will be assessed with calculation of sensitivity, specificity, positive predictive value, negative predictive value, and accuracy (e.g., applying ROC analyses).

To analyse the extent to which the treatment recommendations given match published clinical categorization and treatment guidance (Berk et al. 2007; Kapczinski et al. 2009; Leopold et al. 2013b; Schneck et al. 2017) Cohen’s kappa statistics are applied.

## Discussion

Early-BipoLife is a naturalistic prospective-longitudinal observational cohort study of 1419 participants who are repeatedly assessed during their most vulnerable and formative years (Leopold et al. 2014; Pfennig et al. 2012b; Lish et al. 1994). This comprehensive design has been developed in order (a) to determine the natural course of potential risk and resilience factors and early symptoms/precursors of BD; (b) to observe the change in severity of early symptomatology of BD as primary outcome (including conversion to manifest BD); (c) to evaluate the predictive power of potential risk factors/precursors of BD and biomarkers as well as psychometric properties of recently introduced instruments for the early identification of persons at-risk for BD; and (d) to investigate currently recommended treatment advices in daily care and compare these with recently proposed clinical categorization and treatment guidance (Berk et al. 2007; Leopold et al. 2012; Kapczinski et al. 2009; Schneck et al. 2017).

To the best of our knowledge, this is the first investigation of a help-seeking cohort of at-risk persons for BD sampled from early recognition centres/facilities as well as in-/outpatient settings. Other research on risk factors for BD mainly focused on the investigation of offspring of persons with manifest BD (Duffy et al. 2007, 2009). For a discussion on differences of studies regarding samples, focus, outcomes and statistical approaches see (Geoffroy and Scott 2017).

Major strengths of the Early-BipoLife study are:

- The sample size (N = 1419) and the high number of assessment points during the particular vulnerable phase of disease development and potential conversion to BD. This design allows for the investigation of the natural course of BD from early signs/precursors and subsyndromal symptomatology to research-facilitated diagnosis of manifest BD.
- The reference sample selected from the German representative IMAGEN cohort provides the opportunity to compare the frequency of the proposed risk protective factors in the risk groups with a non-risk cohort. Additionally, data from the previous IMAGEN prospective assessments at age 14 and 16 can be used to analyse courses with and without development of BD symptomatology.
- The assessment of high-risk criteria for BD, potential predictors and subsyndromal symptomatology (e.g., subthreshold depressive and (hypo-) manic symptoms) using recently published instruments in parallel (Bechdolf et al. 2012; Leopold et al. 2012; Correll et al. 2014b). Those standardized diagnostic assessments are repeatedly conducted face-to-face along with a broad range of dimensional measures (on severity and further relevant factors for the natural course of BD).
- The clinical benefit of the supplementation of clinical diagnostics with information on biomarkers is explored.
- Individual resources, resilience factors and their impact on the development, and manifestation (e.g., age of onset) as well as the course of disease is investigated.
- Results from diagnostic assessments and treatment recommendations are comprehensively discussed in a consensus board of clinical experienced staff members (psychiatrists and psychotherapists).
- Data on treatment recommendations and on the effectiveness of applied treatments to patients at risk for BD are investigated in a non-interventional clinical settings. A recently published clinical categorization and treatment guidance/staging model will ideally be refined according to the results on the real-world effectiveness of the treatments applied as part of usual care within the study.

The study has the following limitations. First, this is a naturalistic follow-up study. As such, we will not be able to test the effectiveness of individual components of the treatments that were recommended and delivered. We consider this a second-generation question for further efficacy studies and wish to focus on more generalizable outcomes to study risk and protective factors for BD without the restrictions of an efficacy design and restricted treatment approaches. Second, adherence to the prescribed treatments is not assessed. The study sample includes patients with depression, ADHD, as well as subjects at increased risk for the development of bipolar disorder. In some of these, psychotropic medication is applied out of which the risk for mania might arise. Individual medication is assessed at baseline and FU and will be attended to in the analysis as effectively as possible. Third, we include mostly university centres and hospitals with specialized BD services. While this approach reduces the generalizability of the findings, it provides a yardstick against which other more usual care settings can be compared. Fourth, the study is conducted in Germany and under German health care conditions, with less generalizability of the pathways to care and treatment approaches as well as related illness trajectories in systems with less access to care. Fifth, not all patients enrolling in the Early-BipoLife study will have a complete neuroimaging, electrophysiology and genetics battery. However, given the large number of expected participants, the generated data will be highly informative. We acquire data on somatic diseases and BMI, but no further information on the subject`s physical health. Sixth, the study sample includes patients with depression, ADHD, as well as subjects at increased risk for the development of bipolar disorder. In some of these, psychotropic medication is applied out of which the risk for mania might arise. Individual medication is assessed at baseline and FU and will be attended to in the analysis as effectively as possible. Seventh, with applying a comprehensive FU assessment, especially hypomanic episodes could be detected that otherwise had been overlooked, or had not been classified as such with coarser questioning. Eighth, the control sample is representative for the young population in Dresden and Berlin, was, however, recruited under different conditions as the risk group subjects, which might affect comparability.

Nevertheless, despite these limitations, the Early-BipoLife study will be one of the largest prospective studies to comprehensively characterize a sample of adolescents and young adults at risk for the development of BD. Results are expected to inform patients, families, clinicians, guideline developers, payers and policy makers alike.

Long-term follow-up beyond the visits predefined in the study is planned to increase confidence in the evaluation of the proposed risk and protective factors and lead to a deeper understanding of early stages of BD. At present, all study centers follow patients up to month 36, an extension study will be applied for at a public sponsor.

## Conclusions

The Early-BipoLife study will provide unprecedented and detailed information about the relationship between recently discussed risk and protective factors and the onset and natural course of BD. Findings expected from the Early-BipoLife study will significantly contribute novel insights into pathomechanisms of disease and beneficial treatment algorithms. This information is crucial to further develop early-targeted primary and secondary prevention and intervention strategies to reduce the risk for conversion to BD and improve outcomes when BD has developed. A validated clinical categorization and treatment guidance/staging model will be able to inform patients, clinicians and researchers additionally about the prognosis and treatment response. It is also of particular interest, which assessment tool(s) or which parts of those will perform best to allow a valid categorization and staging of BD risk.

## Data Availability

Data sharing is not applicable to this article as it is a study protocol and no datasets were analysed at this stage of the study process. When publishing the data, the datasets used and/or analysed during the study will be available from the corresponding author on reasonable request.

## Abbreviations

ADHD: Attention-deficit/hyperactivity disorder
ANOVA: Analysis of variance
ASRM: Altman Self-Rating Mania Scale
BAR: Bipolar at risk
BD: Bipolar disorder
BIS: Barratt Impulsiveness Scale
BIS/BAS: Behavioral Inhibition System and Behavioral Activation System
BL: Baseline visit
BPSS-P: Bipolar Prodrome Symptom Scale-Prospective
BWAS: Barron Welsh Art Scale
CAQ: Creative Achievement Questionnaire
CRF: Case Report Form
CTQ: Childhood Trauma Questionnaire
DGBS: German Association of Bipolar Disorders
DGPPN: German Association for Psychiatry and Psychotherapy and Psychosomatics
EEG: Electroencephalography
EPI*bipolar*: Early Phase Inventory for Bipolar Disorders
FAST: Functioning Assessment Short Test
FERUS: Questionnaire for resources and self-management skills
fMRI: Functional Magnet-resonance-tomography
FU 1: Follow-up 1 visit (6 months)
FU 2: Follow-up 2 visit (12 months)
FU 3: Follow-up 3 visit (18 months)
FU 4: Follow-up 4 visit (24 months)
FU 5: Follow-up 5 visit (36 months)
GAF: Global Assessment of Functioning Scale
GCP: Good clinical practice
IDS-C: Inventory of Depressive Symptomatology-Clinician
IPSRT: Interpersonal and social rhythm therapy
IR: Interviewer-rating
MBCT: Mindfulness-based cognitive therapy
MLEQ: Modified Life Events Questionnaire
MRI: Magnet-resonance-tomography
PQ-16: Prodromal Questionnaire
QIDS-SR16: Quick Inventory of Depressive Symptomatology-Self Rating
RG: Risk group
ROC: Receiver operating characteristic
SCID-I: Structured Clinical Interview for DSM-IV-TR Disorders
SCID-II: Structured Clinical Interview for DSM-IV-TR Axis II Personality Disorders
SIPS/SOPS: Structured Interview for Prodromal Syndroms, German Version
SPI-A: Schizophrenia Proneness Interview—Adult version
SR: Self-rating
TEMPS-A: Temperament Evaluation of Memphis, Pisa, Paris and San Diego—Autoquestionnaire short version
TICS: Trier Inventory for Chronic Stress
ToM: Theory of Mind
WHOQOL-BREF: World Health Organization Quality of Life—Short Form
YMRS: Young Mania Rating Scale
